# Neural oscillations are a start toward understanding brain activity rather than the end

**DOI:** 10.1371/journal.pbio.3001234

**Published:** 2021-05-04

**Authors:** Keith B. Doelling, M. Florencia Assaneo

**Affiliations:** 1 Institut de l’Audition, Institut Pasteur, Paris, France; 2 Instituto de Neurobiología, Universidad Autónoma de México Santiago de Querétaro, México

## Abstract

Does rhythmic neural activity merely echo the rhythmic features of the environment, or does it reflect a fundamental computational mechanism of the brain? This debate has generated a series of clever experimental studies attempting to find an answer. Here, we argue that the field has been obstructed by predictions of oscillators that are based more on intuition rather than biophysical models compatible with the observed phenomena. What follows is a series of cautionary examples that serve as reminders to ground our hypotheses in well-developed theories of oscillatory behavior put forth by theoretical study of dynamical systems. Ultimately, our hope is that this exercise will push the field to concern itself less with the vague question of “oscillation or not” and more with specific biophysical models that can be readily tested.

## Introduction

Neural oscillations are a critical phenomenon in neuroscience. For a century, their implication and role have been studied in nearly every cognitive domain and species. Why is research on oscillations so pervasive throughout the field? First, because oscillatory behavior seems to be one of those exceedingly illusive universals of brain function. While these rhythms may occur at different timescales, their presence across brain regions and species is more the rule than the exception [1]. Second, oscillatory dynamics are extremely well studied. Engineers and physicists have studied oscillators to build and understand physical phenomena long before neuroscientists grew interested in them [2,3]. A close look at their work could guide neuroscience toward an explanation of neural function and its consequences.

Despite the potential advantages, it seems that at least a portion of the field is stuck in a loop, debating whether neural oscillations are or are not a useful concept worthy of study. On the one hand, there is a tendency to doggedly look for (and often find) oscillations everywhere (perhaps even where they are not). On the other hand, there exists a reactive trend invoking words like “epiphenomenon,” “evoked response,” and “exhaust fumes” which seeks to explain away any oscillative finding (perhaps even when they are). The result is that more effort is spent on debating an oscillation’s presence than profiting from its advantages. It is our feeling, as researchers who have certainly contributed to this debate, that the discussion no longer provides any tangible benefits to the field. This is because it focuses the field on determining the presence of an oscillation as being a satisfying end goal of a research program, while, instead, it is only a starting point.

An example of this kind of dialog persists in the domain of speech and sound perception. In this field, a wealth of data [4–9] provides evidence for rhythmic activity approximately 4 Hz in the auditory cortex that tracks the rhythms in sound. These papers propose a neural oscillator in the auditory cortex entrained to acoustic stimuli to support a number of important cognitive functions in audition such as attention [10], prediction [11], and segmentation [12]. And yet, the input itself is rhythmic. Perhaps the data can be explained by a passive system whose seeming rhythmicity only reflects the rhythm of the input [13,14]. This passive system relies on some added mechanisms (such as the contingent negative variation, e.g., [15]) to support higher-order processes. What predictions can we use to tease these hypotheses apart, particularly, when the predicted neural recordings are so similar?

We suggest that the question itself is ill-posed. Oscillations may be generated by any number of means. Presumably, the goal of our research is to study not the phenomena but their underlying mechanisms. A basic analysis of how oscillations are generated will reveal 2 important points: (1) the line between oscillation and not is blurred—a leaky integrate-and-fire model is both an oscillator and a model of evoked responses—and (2) there is great heterogeneity in their possible mechanisms, each leading to different behaviors and predictions.

Our goal is not to decide whether oscillators do or do not play a critical role in brain function nor to discuss the advantages of such a mechanism. Instead, we seek to reground ourselves in dynamic systems theories that have studied oscillations in detail and to ensure that the predictions and assessment criteria that we agree upon as a field are adequately justified. We do so by first providing a grounded definition of an oscillator, both generally and by means of simple mathematical equations that can be used to generate valid predictions of its behavior. We then use this mathematical model to test (and often reject) certain commonly assumed predictions. Finally, we provide a framework for refocusing questions away from the presence of an oscillation and toward an ever greater specificity of the kinds of nonlinear dynamics which could yield the neural and behavioral data that we study.

## What is an oscillator?

The word “oscillator” is widely used but loosely defined in the cognitive neuroscience field. Using ambiguous nomenclatures to extensively describe different behaviors is far from optimal. Instead, we propose to redirect our efforts to understand the mechanisms underlying such behaviors by building biophysical models capable of explaining—and/or predicting—experimental observations. For the sake of this argument, we define an oscillator as “a system capable of generating sustained rhythmic behavior by itself.” Here, we refer to a system in the dynamical sense, meaning that the behavior of a set of variables can be described by a set of differential equations adjusted by a set of parameters. Therefore, acknowledging that different parameters may yield different behaviors, we include systems for which self-generating rhythmic behavior occurs within a restricted parameter space. Lastly, we highlight that the oscillator can generate the rhythm by itself; note that we preclude, therefore, a linear system which only oscillates when it receives rhythmic input.

**Fig 1:**
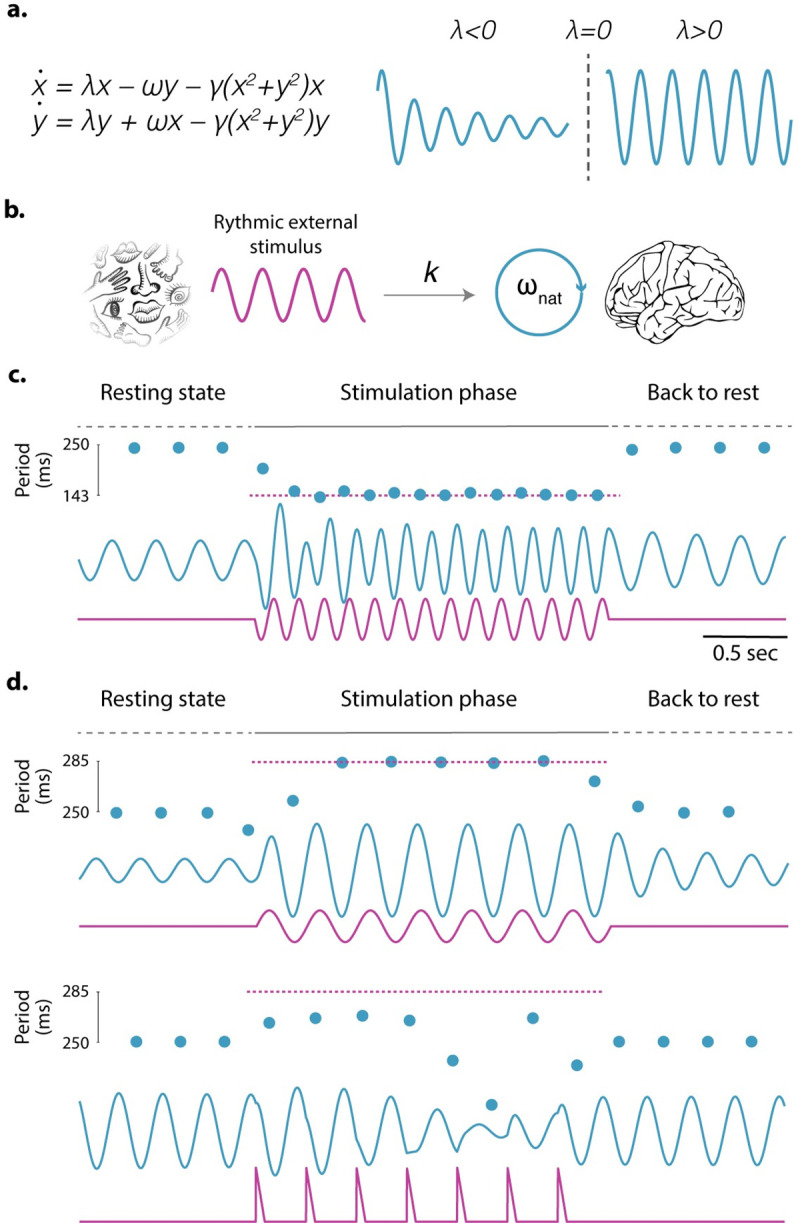
A neural oscillator can be represented by an HA bifurcation. **(a)** The set of equations displayed in the figure, known as Stuart–Landau equations, goes through an HA bifurcation at λ = 0. For λ below this critical value, x(t) (blue trace on the left) shows a damped oscillatory regime; when λ surpasses 0, x(t) (blue trace on the right) starts oscillating with a frequency of *ω*. **(a)** Schematic representation of the oscillator driven bottom-up by an external rhythmic stimulus. Simulations are run for a neural oscillator with a natural frequency *ω*_nat_ = 4 Hz forced by a periodic external stimulus of period *τ*, s(t) = s(t + *τ*). The external stimulus is added as an extra term in the equation driving *x’s* dynamics. The set of equations in panel **a** is modified as $\dot{x}=\lambda x-\omega_{nat}\;y-\gamma (x^{2}+y^{2})+k\;s(t)$. The parameter *k* represents the strength of the coupling between the neural oscillator and the external stimulus. **(c and d)** Oscillator’s response (x(t), blue traces) when forced by different rhythmic stimuli (magenta traces). Blue dots: time evolution of the oscillator’s period. Magenta dashed line: period of the external stimulus. For all simulations, parameters were fixed at *ω_nat_* = 2*π* 4, *λ* = 0.1 and *γ* = 1. For panel **c**, *k* = 25 and *s(t)* a sinusoid with a frequency fixed at 7 Hz. For panel **d**, *k* = 50 and *s(t)* is a sinusoid, for the upper panel, and a periodic train of triangle pulses for the lower one, both with a frequency fixed at 3.5 Hz. HA, Hopf–Andronov.

From a mathematical perspective, there are 4 different ways in which a two-dimensional system begins, or ceases, to oscillate—for a detailed analysis on how oscillations can arise, we invite the reader to study more detailed and complete analyses, e.g., [16,17]. Here, we will only focus on the most famous one: the Hopf–Andronov (HA) bifurcation [16]. In a set of equations describing the dynamics of 2 coupled variables, an HA bifurcation takes place when, by continuously increasing or decreasing a parameter, the system switches from a damped oscillatory regime to sustained oscillations. For example, in the equations shown in Fig 1A, this qualitative change in behavior takes place at λ = 0: For λ<0, the system decays to a stable equilibrium, while for λ > 0, it keeps oscillating.

These equations comprise the Stuart–Landau model, which represents the simplest mathematical description of an HA bifurcation, referred to in the dynamical systems field as a normal form. By applying the correct mathematical manipulations, any system that goes through an HA bifurcation can be reduced (within a region of parameter space close to the bifurcation point) to this normal form.

Others have provided a more comprehensive and detailed description of the Stuart–Landau model than we could hope to achieve [18,19]. Therefore, in the following sections, we will use these equations to illustrate part of an oscillator’s behavioral repertoire. We will show that oscillator behavior should not be constrained by beliefs about how it should behave but rather by a specific mathematical instantiation of the system. For this reason, we have framed the next section in terms of what an oscillator is not, in order to refute commonly held beliefs. In some cases, a more accurate statement might be something akin to “what an oscillator does not have to be,” whereas other rules are more strict. There may well be certain conditions, equations, or parameters under which some of these commonly held beliefs are true. In that case, the authors who put forth these claims must clearly state what these conditions are to clarify exactly what hypotheses are being tested.

## What an oscillator is not

First, some disclaimers. We do not mean to suggest that the points highlighted below are entirely our own. Many other researchers in neuroscience and dynamical systems have discussed these points previously in detail. Some readers may find these points to be trivial, others, highly controversial. Our aim is to counteract a growing trend in the field to deviate away from these tenets and reground the search for neural mechanisms in perception into quantifiable predictions. In what follows, we present some concrete examples on how common beliefs, not grounded on precise biophysical models, can lead to misleading interpretations. We hope it will inspire future research to assess the downstream consequences of specific biophysical models on cognition.

### Oscillators are not echos

Previous work [20,21] suggests that strong evidence for an oscillator requires not only rhythmic activity during stimulation but also after. In line with this proposal, a number of studies have looked for “ringing out” effects where the frequency modulation persists beyond the stimulation period [22–27]. However, the behavior of an oscillator (as for any dynamic system) depends on its current state, on its own dynamics, and on the interaction with upcoming inputs. Which is all to say that it is not necessarily dependent on the frequency of past inputs.

Here, we show that the expectation that an oscillation should ring out at the same frequency as previous stimulation for several cycles after a stimulus input has turned off is not justified—even though oscillator behavior is often tested in such a way. We adopted a model in which the neural oscillator, represented by the equations in Fig 1A, receives rhythmic stimulation as input (Fig 1B). Simulations run with such a model demonstrate that the oscillator returns to its natural state as soon as the external stimulation ceases, without any of the expected reverberant behavior (Fig 1C).

**Fig 2:**
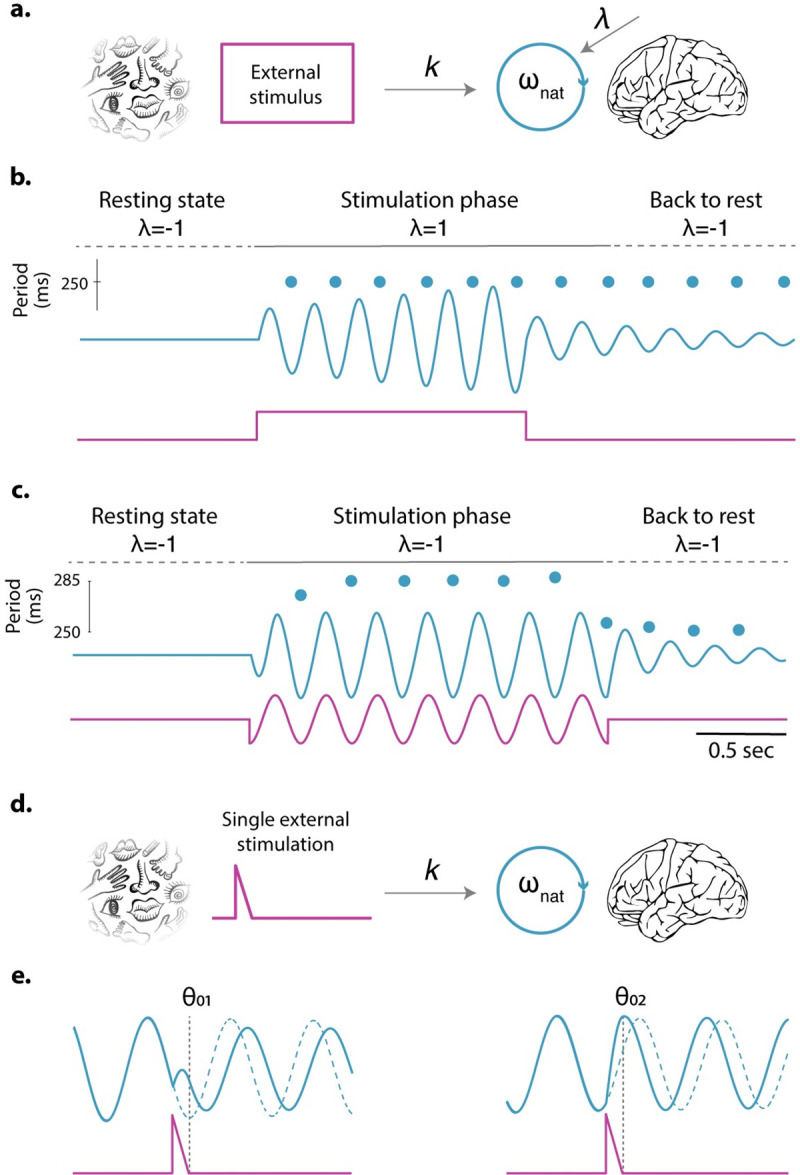
Simulations for different interactions of the neural oscillator with an external stimulus. **(a)** Schematic representation of the model used to run the simulations shown in panels **b and c**: The neural oscillator is driven top-down and/or bottom-up by an external stimulus. The model assumes the same bottom-up interaction as in Fig 1, but this time, it can be modulated by a top-down mechanism. Mathematically, it implies that *λ* is shifted by the presence of an external input. **(b)** The oscillator receives a constant external input, and *λ* is top-down modified by the onset of the stimulus. Simulation parameters are *ω_nat_* = 2*π* 4, *γ* = 1, *k* = 50, and *s(t)* a step function switching from 0 to 1. **(c)** The oscillator is only bottom-up driven by a rhythmic stimulation. Simulation parameters are *ω_nat_* = 2*π* 4, *γ* = 1, *k* = 25, and *s(t)* a sinusoid is a sinusoid with a frequency fixed at 3.5 Hz. **(d)** Schematic representation of the model used to run the simulations shown in panel **e**: The neural oscillator is driven bottom-up by a single event. **(e)** Oscillator’s response when forced by a single stimulus. Once the external perturbation ceases, the oscillator resumes its natural ongoing activity but with a phase lag. The same stimulus resets the ongoing oscillation to different values (*θ*_01_, *θ*_02_) depending on the initial phase. Dashed lines: unperturbed oscillator’s activity. Simulation parameters are *ω_nat_* = 2*π* 4, *γ* = 0.1, and *k* = 50. Blue traces: oscillator’s response, i.e., x(t). Magenta traces: external stimulus, i.e., s(t). Blue dots: time evolution of the oscillator’s period.

From the perspective of the cognitive neuroscientist then, we should not expect an oscillator, at least not in its simplest form, to predict much beyond one cycle after stimulus offset, unless the stimulus is already very close to the oscillator’s spontaneous frequency. If we expect a neural mechanism to extend for many cycles beyond this, more specifics are required regarding the nature of the particular oscillator.

Many papers have sought out “ringing out” effects, and not all of them are out of line in doing so. By virtue of receiving an external input, the phase of any oscillator will most likely be shifted by some nonzero amount, and therefore will be altered by the input. This phase shift could result in higher intertrial phase coherence several cycles after the stimulus ceases. Indeed, some studies have found shifts in phase on this basis [28–30]. However, as Fig 1C shows, poststimulus, the oscillator reverts to its intrinsic (spontaneous) frequency. Therefore, searching for phase shifts at the stimulus frequency will lead to subpar results.

### Oscillators are not shape invariant

This point has not often been considered in perceptual neuroscience, but it is an important one if we are to test the viability of an oscillator as a perceptual principle. In general, when considering how an oscillator might synchronize to a rhythmic input, one often assumes that the behavior should be similar across many different input shapes: whether the signal is a smooth violin note, a sharp pure tone, or something in between like speech. However, this feature has not been considered in enough detail regarding how it should affect the neural ability to synchronize to it. And yet, it can make a big difference.

For example, consider the simulations run with the same model as in the previous subsection, which show that the neural response depends on the specific shape of the rhythmic stimulation (Fig 1D). When the system is forced with a sinusoidal stimulus, it immediately synchronizes to the external frequency. However, when the stimulus has the same period (i.e., frequency) and max amplitude but a pulsatile shape, the entrainment becomes unstable. We should note here that these simulations represent only an example and that the sinusoidal stimulus is an arbitrary choice. A pulsatile stimulus can also generate stable synchrony, particularly if it is closer to the input’s natural frequency. Similarly, some sine waves, depending on their frequency, may also generate unstable behavior. One should conclude from these simulations that oscillatory behavior is not invariant to input shape and that its response will depend heavily on the oscillator’s phase response curve: the amount of phase shift produced by a unit of input as a function of the ongoing phase (see “Oscillators are not phase invariant” for more details).

From the cognitive perspective, the study of how stimulus shape affects neural synchronization and temporal perception is understudied—however, see work on steady-state visual evoked potentials [31–33]. Any theoretical models of oscillatory synchrony to perceptual input should also take these dynamics into account, perhaps by defining some preprocessing steps which might normalize the input shape before it is fed into the oscillator. For example, previous works in the auditory domain have considered such preprocessing, showing that the sharpness of the acoustic envelope, also called acoustic edges, may be a key feature represented in the auditory cortex [34–36].

### Oscillators are not tireless

Using the definition of an oscillator that we began with (a system that is capable of self-generating oscillations), we can see quickly that consistent spontaneous oscillations are not a requirement. In general, dynamic models of neural function are often studied near bifurcations whereby a small shift in the parameters can create topologically distinct behaviors [37,38]. In that sense, a system which starts out at rest can reach a limit cycle (i.e., an oscillatory regime) during stimulation, which is then destroyed when the stimulus is turned off. The capacity to oscillate does not require the oscillation to continue endlessly.

From a neural perspective, brain regions are not isolated; it is through their interaction that a neural system reaches complex behaviors. Thus, a valid assumption is that some of the parameters in the equations in Fig 1A could be modulated through the activity of other brain areas and/or by presence or absence of the stimulus itself (see Fig 2A). For example, a top-down mechanism could push the system across a bifurcation point, bringing the system into an oscillatory regime when it detects an external input. As soon as the stimulus disappears, the system goes back to its non-oscillatory regime by progressively decreasing the oscillation’s amplitude back toward rest (see Fig 2B).

Alternatively, from a bottom-up perspective (i.e., for a fixed *λ* value) a rhythmic external input can also elicit oscillations from the system. In contrast to the previous example, where the system oscillates at its natural frequency, here, during the stimulation phase, the frequency is determined by the rhythmic features of the stimulus (see Fig 2C). Once the stimulation ceases, the system goes back to rest, oscillating at its natural frequency and progressively decreasing its amplitude toward zero.

All in all, these simulations exemplify how, through different mechanisms, a quiescent system at rest can turn into an oscillatory regime during stimulus input and back again.

The implication here then is that studies searching for ongoing fluctuations prior to the stimulus as proof of an oscillator may indeed be misguided. While the presence of rhythmic fluctuation in the baseline period may well be indicative of oscillatory behavior, the absence of these fluctuations does not indicate the absence of an underlying oscillator. Instead, oscillatory behavior should be defined by how the mechanism interacts with the stimulus while the stimulus is present, as we have sought to do in this essay.

A corollary of the above is that stimulus input can change oscillator amplitude. The belief that an oscillator must constantly oscillate could invite the prediction that oscillators will maintain the same amplitude before and during stimulation and only shift in phase. Therefore, a change in amplitude would be indicative of an evoked response. Each of our simulations refute this concept: Amplitude change with stimulus input is a common feature of oscillatory behavior.

### Oscillators are not phase invariant

An oscillator does not respond uniformly even to the same input at different moments in time. How an oscillator’s phase shifts in response to input is a function of its ongoing phase: a phenomenon typically known as the phase response curve or phase resetting curve (PRC; see [39]. The PRC has been considered at the level of circadian rhythms (e.g., [40,41]) and of single cell and circuits (e.g., [42]) but remains understudied at the human cognitive level.

Proposed oscillations, as presented in cognitive science papers, often ignore this point, assuming for the sake of simplicity that each input adjusts the phase by the same amount, or not at all, (e.g., [20] Fig 1; [43] Fig 1; [44] Fig 1A) or that the phase will always be totally reset to 0 (e.g., [23] Fig 1B; [45] Fig 1). Many of these examples present oscillators in this fashion as simplifications for didactic purposes. We do not suggest that the authors are unaware of this phenomenon. However, we feel that these simplifications are in danger of being taken too literally. The sensitivity of the PRC can have important consequences to the adaptability of an oscillator. The degree (and the direction) to which the phase is modified depends on the phase at the time of the arrival of the stimulus. Critically, an oscillator’s phase response function will depend on the specific oscillator. Here, we show again the same exemplar oscillator to illustrate this point.

In Fig 2D and 2E, a single pulsatile stimulus, applied at 2 distinct phases, yields 2 very different phase adjustments. In the first case, by stimulating the oscillator during its downward phase, the oscillator’s “natural progression” is inhibited resulting in a positive phase lag. In the second case, by stimulating the oscillator during its upward phase, the oscillator’s progression is facilitated resulting in a phase advancement. This kind of flexible response is a feature—and not a bug—of the oscillator, because it allows for precise computation based on the timing of the input: The system can speed up or slow down depending on when the stimulus arrives.

### Oscillators are not metronomes

**Fig 3:**
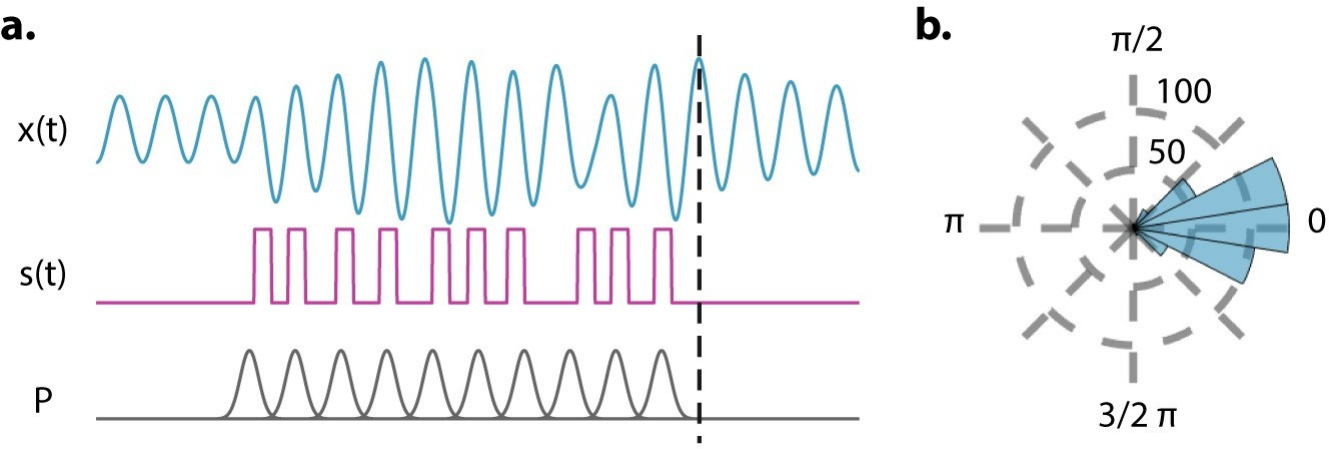
An oscillator can handle some irregularity. **(a)** The output of the Stuart–Landau model (x(t) shown in blue) in response to an example quasi-rhythmic stimulus (s(t) in magenta) that was drawn from Gaussian distributions whose means are centered in a rhythmic fashion (P in gray). Gaussian distributions have a standard deviation of 20% of expected time between tones creating substantial deviations from rhythmicity. The dashed black line shows where the mean of the next Gaussian distribution would be and therefore the expected location of the next tone if there were one. It lines up well with the peak of the oscillator. **(b)** A histogram of the phase extracted at the dashed line in panel **a** over 500 different stimuli. Phase clustering suggests that the oscillator phase contains information regarding timing of expected tones. Simulation parameters are *ω_nat_* = 2*π* 4, *λ* = 1, *γ* = 1, *k* = 10, and *s(t)* is made up of 100-ms square steps with each onset drawn from a distribution in P. P is a set of Gaussians with μ = 0.25a, where a is {0, 1, 2,…, 9} and **σ** = 0.05.

Nikolić and colleagues [46] wrote a spotlight article entitled “Gamma oscillations: precise temporal coordination without a metronome.” We too invoke the metronome metaphor to emphasize a similar point. Oscillations are not sine waves, and their flexibility is a crucial aspect of their utility. To drive this final point home, we created a brief simulation (Fig 3) using the same Stuart–Landau model from the previous simulations to show an example of how the model behaves in response to quasi-rhythmic stimuli. Each unit of stimulation is drawn from a Gaussian distribution, each centered in rhythmic fashion so that the expected locations of each tone would yield a perfectly rhythmic stimulus. The example stimulus shown in Fig 3A demonstrates that despite the deviations in timing of each stimulus from rhythmicity, the oscillator still peaks at the expected time of what would be the next tone.

We extracted the phase at this expected time and ran this simulation 500 times, each with a new stimulus drawn from the same distributions. Fig 3B shows that the oscillator phase at the expected time across these simulations is highly concentrated around 0 and therefore that the oscillator phase carries information about the expected timing of upcoming inputs. In this sense, the oscillator can speed up or slow down depending on the deviations from rhythmicity that it receives.

## Not all that oscillates is oscillator

**Fig 4:**
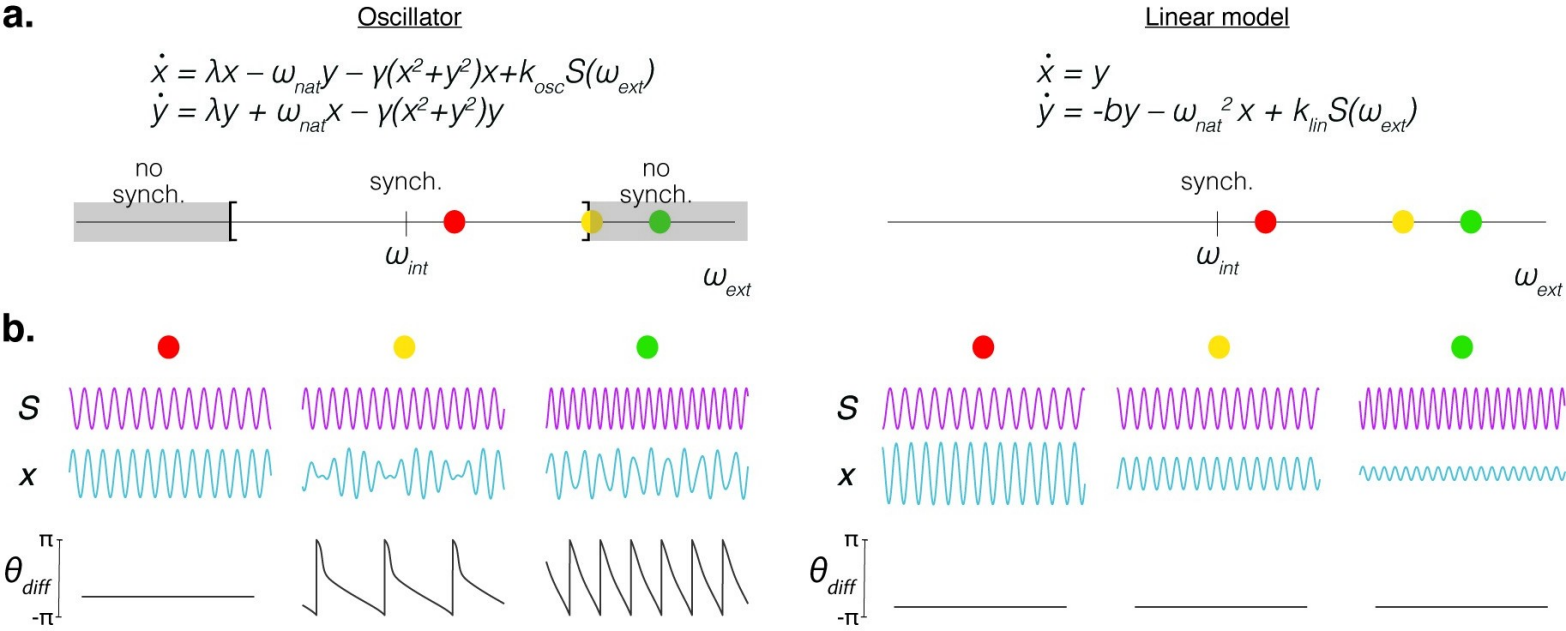
Comparison between an oscillator and a non-oscillator–like systems. **(a)** Upper panel: set of equations defining the different models. An oscillator-like model (Stuart–Landau) on the left; a linear model on the right. Lower panel: system’s behavior for different frequency values (*ω*_ext_) of the external rhythmic signal (*S*). For the oscillator, the synchronization between the system and the external force vanishes when the external frequency departs from the internal one (gray area in the left panel); for the linear model, instead, synchrony exists regardless of the value adopted by the external frequency. **(b)** Behavior of the 2 models (blue traces; oscillator on the left, linear model on the right) for rhythmic inputs (magenta traces) of different frequencies (colored dots; red, yellow, and green correspond to *ω*_ext_ = 4.5, 5, and 6.2 Hz, respectively). The lower panel displays in gray the time evolution of the phase difference between the system output and the corresponding input.

The above examples show the potential and flexibility of oscillators, but their bounds are not limitless. Here, we run some simulations to exemplify how an oscillator can be distinguished from a non-oscillator system. For the oscillator, in line with the previous sections, we adopted the Stuart–Landau model. For the non-oscillator, we chose a linear damped system. The linear system is more akin to a resonant system which has a preferred frequency of stimulation but is not self-generative (see [21] for a detailed description of a resonant system). One divergence between the models’ predictions is that, when forced with an external rhythmic signal, the linear model follows the external rhythm regardless of the forcing frequency (with reductions in amplitude as the stimulus frequency moves further away from the preferred), while the oscillator, instead, synchronizes only if the forcing frequency is within a range around its natural rhythm (Fig 4A). To distinguish between these quantitatively different behaviors, a useful analysis is to compute the phase difference between the output of the system and the forcing signal (Fig 4B). If the empirical observation shows that when presented with an isochronous stimulus the phase difference between the stimulus and the brain signal remains constant, regardless of the external rhythm, the simplest model should be adopted. Since the linear model predicts the observations, there would be no justification to increase the complexity of the model by adding a nonlinearity. In this direction, we can point to several studies assessing the oscillator-like properties of the neural system using this kind of analysis [47,48].

In general, the behavior of a neural oscillator will depend on the specific features of the dynamical system defining it and on the mechanisms through which it interacts with the external world—bottom-up, like Fig 1D, and/or top-down, like Fig 2A, where an internal parameter of the oscillator is modified by the external stimulus.

## Not all oscillators are alike

Each oscillator oscillates in its own way, the way that each nonlinearity is nonlinear in its own way. Therefore, the claim that some neural system oscillates is akin to calling the system nonlinear: useful information but hardly a complete explanation. More specifics are required to meaningfully advance the field forward. Conversely, by ruling out any of the behaviors discussed here, an experimentalist has constrained the kind of oscillator, or other dynamical system, that could have performed this function. This is an important contribution. Still, we caution the field against ruling out one behavior and throwing out all possible oscillators along with it. Doing so may ultimately harm progress in the field.

How then should the field move forward? We believe that rather than concerning ourselves with supporting or refuting neural oscillators, a more useful framework would be to focus our attention on the specific neural dynamics we hope to explain and to develop candidate quantitative models that are constrained by these dynamics. Furthermore, such models should be able to predict future recordings or be falsified by them. That is to say that it should no longer be sufficient to claim that a particular mechanism is or is not an oscillator but instead to choose specific dynamical systems to test. In so doing, we expect to overcome our looping debate and to ultimately develop—by means of testing many model types in many different experimental conditions—a fundamental understanding of cognitive processes and the general organization of neural behavior.
